# Who sleeps under bednets in Ghana? A doer/non-doer analysis of malaria prevention behaviours

**DOI:** 10.1186/1475-2875-5-61

**Published:** 2006-07-25

**Authors:** Natalie De La Cruz, Benjamin Crookston, Kirk Dearden, Bobbi Gray, Natasha Ivins, Stephen Alder, Robb Davis

**Affiliations:** 1 Department of Health Science, Brigham Young University, Provo, UT, USA; 2 Freedom from Hunger, Davis, CA, USA; 3 Department of Family and Preventive Medicine, University of Utah, Salt Lake City, UT, USA; 4 Mennonite Central Committee, Akron, PA, USA

## Abstract

**Background:**

Malaria prevention programmes should be based in part on knowledge of why some individuals use bednets while others do not. This paper identifies factors and characteristics of women that affect bednet use among their children less than five years of age in Ghana.

**Methods:**

Data come from the baseline component of an evaluation of Freedom from Hunger's malaria curriculum. A quasi-experimental design was used to select clients (n = 516) of *Credit with Education *(an integrated package of microfinance and health education) and non-clients (n = 535). Chi-squares, Fisher's Exact tests and logistic regression were used to compare the characteristics of mothers whose children use bednets (doers) with those whose children do not (non-doers) and to identify factors associated with bednet use among children less than five years of age.

**Results:**

The following factors were most closely associated with bednet use: region of residence; greater food security; and caregivers' beliefs about symptoms, causation and groups most vulnerable to malaria. Most respondents knew mosquitoes caused malaria; however, 20.6% of doers and 12.3% of non-doers (p = .0228) thought overworking oneself caused malaria. Ninety percent of doers and 77.0% of non-doers felt that sleeping under a net was protective against malaria (p = .0040). In addition, 16.5% of doers and 7.5% of non-doers (p = .0025) identified adult males as most vulnerable to malaria.

**Conclusion:**

Greater knowledge about malaria does not always translate into improved bednet use. Though culturally-based ideas about malaria may vary between communities, integrating them into traditional health education messages may enhance the effectiveness of public health efforts.

## Background

Malaria threatens the lives of 3.2 billion people globally and leads to over one million deaths annually [1]. Malaria is hyper-endemic in Ghana, accounting for 44% of outpatient attendance, 13% of all hospital deaths, and 22% of mortality among children less than five years of age [1,2]. Though malaria is responsible for 9% of overall mortality in Ghana, at least 40% of malaria deaths occur among infants and children under the age of five [3].

The Roll Back Malaria campaign emphasizes the use of insecticide-treated bednets (ITNs) which are effective in reducing human contact with mosquitoes [1]. Results of recent research suggest that ITNs can reduce malaria episodes by 48–50% [4] and, if universally used, could prevent an estimated seven percent of global under five mortality [5]. The use of ITNs could save the lives of more children than any other single intervention except breastfeeding and oral rehydration therapy [5]. At an estimated cost of US$1.2 per person protected per year, ITNs are considered to be one of the most cost-effective health interventions in Ghana [6].

Despite evidence demonstrating that the use of ITNs decreases malaria-related morbidity and mortality, it is difficult to encourage consistent and appropriate use of bednets. Estimates from Africa as a whole suggest that only three percent of children less than five years of age sleep under ITNs, while up to ten times as many are thought to sleep under any bednet [1]. Past research in a variety of countries reveals that children may fail to sleep under bednets for a number of reasons. For example, parents attribute malaria to causes other than mosquitoes and may not associate bednet usage with the prevention of malaria. Additionally, parents may consider it too costly to purchase a bednet or to treat it with insecticide and, despite increasing availability and improved distribution systems, many communities have limited access to bednets [1,7]. Parents may consider using bednets difficult because sleeping under them can be hot and uncomfortable; or, they may believe that bednets resemble a burial shroud or that insecticides used to treat ITNs will harm their children [8]. On the other hand, community members often value bednets because they decrease the nuisance associated with mosquitoes. However, such beliefs often undermine the consistent use of bednets during the dry season when mosquitoes are less noticeable [9-11].

Many other factors also influence caregivers' decisions and malaria prevention behaviours [12,13]. For example, community perceptions, beliefs and attitudes about malaria causation, prevention and cure influence efforts to address malaria and are often overlooked in control efforts [14,15]. In the case of Ghana, some individuals maintain the notion that certain types of "fever" (local terms for fever and malaria often coincide) are caused by the heat of the sun and, therefore, cannot be prevented by bednet use, intermittent treatment or insecticides [16]. Cost may also discourage parents from practicing optimal health behaviours. With malaria, the cost of bednets and the cost of treatment (clinic fees and medications) for sick individuals play an important role in determining which behaviours parents practice. In addition, the incidence of malaria and immunity levels within a population can influence community perceptions of disease severity and may influence the perceived importance of engaging in behaviours such as bednet use [13,17].

As policy makers and programmers in Ghana seek to improve malaria prevention behaviours, it is critical that they are fully informed of the determinants of such behaviours. One way of improving the effectiveness of malaria prevention efforts is to better understand what distinguishes individuals who practice optimal behaviours ("doers") from individuals who do not ("non-doers"). The purpose of this paper is to identify knowledge-related factors that affect bednet use among children under five years of age. This study uses baseline data from Ghana to test the hypothesis that mothers who practice optimal malaria prevention behaviours by having their child sleep under a bednet (doers) 1) have a better understanding of the cause of malaria as well as appropriate protective methods; 2) are more likely to know the signs and symptoms of malaria; 3) are able to identify those most vulnerable to malaria; 4) know the potential consequences of malaria during pregnancy; and 5) understand how to protect pregnant women from malaria.

## Methods

### Background

Data for this study come from the baseline component of a study designed to assess the impact of Freedom From Hunger's malaria educational model in Ghana. Freedom from Hunger (FFH) – an international non-governmental organization (NGO) – works in sixteen countries around the world. Their mission is to bring sustainable, self-help solutions to communities as they address chronic hunger and poverty and equip families with resources they need to build health, hope and dignity. The study was carried out in 13 communities in the Afram Plains District of the Eastern Region and 20 communities in the Asikuma-Odoben-Brakwa District of the Central Region. The Afram Plains District extends into the Volta Lake Basin and is among the most geographically disadvantaged districts in the country, as it can only be reached by ferries from the southwest and east. The Asikuma-Odoben-Brakwa District is located in the north-central portion of the Central Region and has a somewhat higher population density than Afram Plains. In both study areas, malaria remains the most common reason for attending a health facility.

Freedom from Hunger (FFH) uses *Credit with Education *to offer an integrated package of microfinance services combined with health education. Women within each credit association meet weekly, biweekly, or monthly to repay loans, to deposit savings and to participate in non-formal, dialogue-based learning sessions where they discuss better health, nutrition and family planning practices as well as sound business strategies.

In 2004, prior to introducing a new malaria education module developed for West Africa, FFH carried out baseline data collection as part of the first phase of a project intended to evaluate the impact of the module in Ghana. The purpose of the baseline assessment was to describe community members' knowledge and practices prior to the introduction of malaria education. Data for this study were drawn from the baseline assessment.

### Instrument

The quantitative data collection instrument was adapted from a standardized tool (the Malaria Indicator Survey) developed by RBM partners and was designed to assess core household indicators. Several questions were also adapted from the 2003 Ghana Demographic and Health Survey. Prior to administering the survey, qualitative research was carried out to determine community perceptions of malaria and the survey was adapted accordingly.

Survey respondents included women of reproductive age (15–49) with a child less than six years of age. Each respondent was interviewed about sociodemographic and household characteristics as well as knowledge and practices regarding malaria transmission, prevention and treatment. Variables used for assessing respondents' knowledge included cause of malaria; steps in the transmission cycle; methods of protecting against malaria transmission; best ways to prevent malaria; stopping treatment before a full dose is completed; which groups are most vulnerable to malaria; and signs and symptoms of malaria in children and pregnant women.

### Sample selection and data collection

A complete list of credit association members was used to randomly select clients from the 31 credit associations in the Eastern Region and the 51 credit associations in the Central Region. Prior to selecting clients, credit associations participating in the *Credit with Education *programme were randomly assigned to receive either the malaria or a standard diarrhoea module upon the completion of baseline data collection.

Non-clients were selected from the same communities as clients through two-stage cluster sampling. Each community was divided into four geographic segments, one of which was randomly selected. Non-clients were selected by systematically sampling the houses in the randomly chosen geographic sector. A starting point was randomly selected and every 5th household was then surveyed. The same eligibility criteria (women of reproductive age with a child less than six years of age) were used in the selection of clients and non-clients.

Trained interviewers collected data between September and October of 2004. Selection criteria for interviewers included the ability to speak the local dialects (Ewe, Twi and Fante) in addition to the national language (English); previous experience with data collection; and at least a high school education. Though the questionnaires were in English, the actual interviews were conducted in the local languages and each woman's responses were transcribed by the interviewer. In order to minimize variations between interviewers, during training, interviewers discussed and standardised how key questions would be asked in each language.

Interviewers briefly explained the research objectives and obtained written consent from participants. Because sociodemographic information and several other independent variables were only collected at the household level and because there were, on average, 1.5 women of reproductive age per household, only primary respondents – those women originally selected for interviews or the appropriate replacement that subsequently provided information on the other members of the household – were considered for the current analyses. Therefore, 1,051 women (n = 537 from the Central region; n = 514 from the Eastern region) were included in the analytic dataset, half (49.1%) of whom were clients of *Credit with Education *programmes.

Data from various subgroups of the original sample were analysed; however, this paper focuses exclusively on children less than five years of age. Though about 25% of women had more than one child under the age of five, only the *youngest *child in each household was included for analyses. Thus, the sample used for this study included 900 youngest children under the age of five. Before excluding older children (also less than five years of age) from analyses, it was confirmed that there were no significant differences in the behaviours of interest between the youngest child and other children in the household. Overall, 9.5% of *all *children less than five years of age slept under a bednet the night before the survey while only 1.4% slept under an ITN. Because only a very small percentage of all children under five slept under bednets that were verifiably insecticide treated, ITN use alone was not evaluated. Bednets that have not been treated with insecticide offer some protection against malaria, but the protection provided by bednets is maximized with treatment and regular re-treatment with insecticide [7]. In this paper, bednets refer to *any *bednet – insecticide treated or otherwise.

### Data analysis

Data were analysed using SAS, version 9.1 (SAS Institute Inc., Cary, North Carolina, USA). Frequencies, percentages, Pearson chi-square tests and Fisher's Exact tests were used to compare the sociodemographic characteristics and knowledge of doers with non-doers in relation to bednet use. Differences between clients and non-clients were checked and found to be minimal. Logistic regression was then used to identify those factors most closely associated with bednet use among children less than five years of age. Variables were retained or dropped from the models through stepwise regression based on a 0.2 level of significance. Odds ratios and 95% confidence intervals were calculated for all retained variables.

## Results

Socio-demographic characteristics for primary respondents are shown in Table 1. Most respondents were between 25 and 44 years of age and had completed at least primary schooling. Additionally, nearly 98% of respondents had received professional antenatal care during their last pregnancy. Differences between the two regions were significant for most variables (see Additional file 1).

**Table 1:** Characteristics of Primary Respondents, Central and Eastern Regions, Ghana 2004

Characteristic	N	%
Respondent's Age		
15–24	173	16.5
25–34	457	43.5
35–44	319	30.4
45–49	102	9.7
Region		
Central	537	51.1
Eastern	514	48.9
Credit Association Member		
Yes	516	49.1
No	535	50.9
Education		
Highest level attended		
No School	191	18.3
Primary	245	23.5
Junior Secondary/Middle	533	51.1
Senior Secondary/Vocational/Higher	75	7.2
Household Size		
1–3	140	13.3
4–5	366	34.8
6–7	343	32.6
8 or more	202	19.2
Religion		
Christian	926	89.3
Muslim/Other	111	10.7
Water Supply		
Piped in/Covered well in yard	44	4.2
Public Tap/Borehole	818	77.9
Open Water Source	188	17.9
Toilet Facility		
Public Latrine	606	57.9
Private Toilet/Latrine	324	31.0
Bush	116	11.1
Antenatal Care During Last Pregnancy		
Yes	995	97.9
No	21	2.1
Type of Antenatal Care		
Professional	974	97.9
Non-professional	21	2.1
Food Security		
Food Secure	572	56.1
Food Insecure without Hunger	214	21.0
Food Insecure with Hunger	234	22.9

### Knowledge and practices regarding malaria causation, prevention and control among the overall sample

The majority of respondents in both regions (92.9% in the Central and 97.3% in the Eastern region) identified mosquito bites as a cause of malaria; however, roughly two-thirds of respondents named additional causes as well. Sleeping under an ITN was the most commonly identified protective method against malaria. Using mosquito coils and clearing bushes around the house were also cited as protective, particularly in the Central region where such methods were identified almost as often as ITNs. Nearly 70% of respondents in both regions named fever as a symptom of malaria. Chills – a primary symptom of malaria – were recognized by 27.6% of respondents in the Central region and only 16.0% of respondents in the Eastern region. Children less than five years of age and pregnant women were often identified as the groups most vulnerable to malaria, though additional "most vulnerable" groups were typically mentioned simultaneously.

### Differences between doers and non-doers

#### Bednet use

Socio-demographic and other factors associated with bednet use among children under five are summarized in Table 2. Socio-demographic factors significantly associated with bednet use on the night prior to the interview included region of residence, water source, type of toilet facility and level of food security. More specifically, children who slept under a bednet the night before the interview were more likely to come from the Eastern region and to live in households that obtained their water from an open water source and used a private toilet/latrine or the bush. Children who slept under a bednet were also more likely than those who did not to live in a food secure household.

**Table 2:** Net Use among Children Less than 5, Central & Eastern Regions, Ghana 2004

Characteristic	*Doers*		*Non-Doers*		*p*
	*n = 97*	%	*n = 803*	*%*	

Respondent's age					.2452
15–24	17	17.5	149	18.6	
25–34	50	51.6	358	44.6	
35–44	27	27.8	228	28.4	
45–49	3	3.1	68	8.5	
Live in Eastern region	80	82.5	375	46.7	**<.0001**
Belong to a credit association	41	42.3	386	48.1	.2798
Highest level of schooling attended					.2406
No school	19	19.8	143	17.9	
Primary	26	27.1	185	23.2	
Junior secondary/middle	41	42.7	419	52.4	
Senior secondary/vocational/higher	10	10.4	52	6.5	
Household size					.2192
1–3	16	16.5	110	13.7	
4–5	29	29.9	299	37.2	
6–7	38	39.2	247	30.8	
8 or more	14	14.4	147	18.3	
Religion					.6839
Christian	88	90.7	707	89.4	
Muslim/other	9	9.3	84	10.6	
Water supply					**.0174**
Piped in/covered well in yard	2	2.1	29	3.6	
Public tap/borehole	67	69.1	635	79.2	
Open water source	28	28.9	138	17.2	
Toilet facility					**.0015**
Public latrine	42	43.3	472	59.1	
Private toilet/latrine	34	35.1	241	30.2	
Bush	21	21.7	86	10.8	
Received antenatal care during last pregnancy	95	97.9	776	97.6	.8405
Received professional antenatal care during last pregnancy	93	97.9	758	97.7	.8953
Food security					**.0324**
Food secure	63	66.3	432	55.4	
Food insecure without hunger	20	21.1	158	20.3	
Food insecure with hunger	12	12.6	190	24.4	
Believe mosquito bites cause malaria	94	96.9	765	95.3	.6109*
Believe staying in the sun too long causes malaria	36	37.1	343	42.7	.2912
Believe working too close to fire causes malaria	14	14.4	118	14.7	.9451
Believe overworking yourself causes malaria	20	20.6	99	12.3	**.0228**
Believe in spiritual/superstitious causes of malaria	2	2.1	16	2.0	.9633
Believe in other causes of malaria	19	19.6	163	20.3	.8692
Do not know what causes malaria	1	1.0	13	1.6	.6585
Believe in the correct cause of malaria (mosquito bites only)	22	22.7	247	30.9	.0962
Believe sleeping under an ITN is protective against malaria	87	89.7	618	77.0	**.0040**
Believe mosquito coils are protective against malaria	48	49.5	490	61.0	**.0286**
Believe clearing bushes around the house is protective against malaria	56	57.7	387	48.2	.0759
Believe wearing clothes to cover the body is protective against malaria	7	7.2	72	9.0	.5651
Believe disturbing mosquito hiding places is protective against malaria	8	8.3	65	8.1	.9585
Believe in other protective methods against malaria	8	8.3	132	16.4	**.0355**
Do not know protective methods against malaria	2	2.1	19	2.4	.8513
Believe that the best way to prevent malaria is:					**.0042**
Using an ITN	88	90.7	613	76.6	
Other good alternatives	4	4.1	125	15.6	
Don't know	5	5.2	62	7.8	
Believe fever is a sign/symptom of malaria	70	72.2	555	69.1	.5380
Believe vomiting is a sign/symptom of malaria	62	63.9	449	55.9	.1329
Believe yellowish/dark urine is a sign/symptom of malaria	39	40.2	407	50.7	**.0512**
Believe a lack of appetite is a sign/symptom of malaria	45	46.4	314	39.1	.1661
Believe chills is a sign/symptom of malaria	17	17.5	175	21.8	.3325
Believe diarrhoea is a sign/symptom of malaria	8	8.3	114	14.2	.1059
Believe headache is a sign/symptom of malaria	8	8.3	113	14.1	.1122
Believe joint pain or bone pain is a sign/symptom of malaria	11	11.3	96	12.0	.8597
Believe dizziness is a sign/symptom of malaria	3	3.1	70	8.7	.0731*
Believe constipation is a sign/symptom of malaria	2	2.1	23	2.9	.6497
Believe in other signs/symptoms of malaria	11	11.3	123	15.3	.2986
Believe that a child with fever needs to complete a full course of treatment even if his conditions improve	87	89.7	632	79.3	**.0149**
Believe that children <5 are most vulnerable to malaria	82	84.5	694	86.4	.6100
Believe that children 6–14 years old are most vulnerable to malaria	11	11.3	108	13.5	.5624
Believe that pregnant women are most vulnerable to malaria	55	56.7	470	58.5	.7299
Believe that women of reproductive age (15–49) are most vulnerable to malaria	9	9.3	70	8.7	.8537
Believe that adolescent boys (15–18 yrs) are most vulnerable to malaria	3	3.1	44	5.5	.4674*
Believe that adult males (above 18 yrs) are most vulnerable to malaria	16	16.5	60	7.5	**.0025**
Believe that everyone is most vulnerable to malaria	24	24.7	115	14.3	**.0073**
Do not know who is most vulnerable to malaria	1	1.0	17	2.1	.7104*
Believe that only children <5 years are most vulnerable to malaria	9	9.3	133	16.6	.0630
Believe that only pregnant women are most vulnerable to malaria	1	1.0	22	2.7	.4998*
Believe that only children <5 and pregnant women are most vulnerable to malaria	21	21.7	216	26.9	.2675
Believe that malaria during pregnancy can lead to miscarriage/abortion	63	65.0	494	61.5	.5113
Believe that malaria during pregnancy can lead to death	57	58.8	477	59.4	.9036
Believe that malaria during pregnancy can lead to premature delivery	35	36.1	286	35.6	.9279
Believe that malaria during pregnancy can lead to anaemia	13	13.4	152	18.9	.1839
Believe that malaria during pregnancy can lead to profuse bleeding	13	13.4	123	15.3	.6188
Believe that malaria during pregnancy can lead to something else	4	4.1	35	4.4	.9145
Do not know any consequences of malaria during pregnancy	6	6.2	22	2.7	.1099*
Believe that starting antenatal care once pregnant is protective against malaria during pregnancy	63	65.0	542	67.5	.6135
Believe that sleeping under an ITN is protective against malaria during pregnancy	53	54.6	363	45.2	.0784
Believe that eating good food/a balanced diet is protective against malaria during pregnancy	43	44.3	372	46.3	.7095
Believe that keeping surroundings clean is protective against malaria during pregnancy	40	41.2	232	28.9	**.0124**
Believe that taking antimalarials as directed is protective against malaria during pregnancy	15	15.5	155	19.3	.3616
Believe that treating worm infestations is protective against malaria during pregnancy	3	3.1	41	5.1	.6154*
Believe that something else is protective against malaria during pregnancy	4	4.1	82	10.2	.0653*
Do not know of any ways to protect against malaria during pregnancy	4	4.1	10	1.3	.0544*
Believe that pregnant women can have malaria without knowing or showing signs of it	46	47.9	413	52.5	.3915

*Fisher's Exact

Malaria related beliefs and knowledge were significantly associated with bednet use as well. Doers – caregivers of children who slept under a bednet – were significantly more likely than non-doers to believe that overworking oneself was a cause of malaria and to feel that sleeping under an ITN was an important protective method and/or the best way to prevent malaria. Doers were also significantly more likely to disagree with the statement that a child with fever does not need to complete a full course of treatment if his conditions improve and to identify "everyone" or adult males as most vulnerable to malaria. In addition, compared to non-doers, doers were more likely to believe that keeping surroundings clean was a way to protect against malaria during pregnancy.

Results from logistic regression analyses (Table 3) were similar, though some differences are evident. Doers were 5.6 times more likely than non-doers to believe that dizziness was not a sign or symptom of malaria (95% CI: 1.3, 24.6). Doers were 4.9 times more likely than non-doers to live in the Eastern region (95% CI: 2.6, 9.2) and were 2.5 times more likely to come from food secure households. Additionally, compared to non-doers, doers were approximately twice as likely to identify overworking oneself as a cause of malaria, keeping surroundings clean as a protective measure against malaria during pregnancy, and adult males as the group most vulnerable to malaria (2.0, 1.8 and 2.2 times more likely, respectively).

**Table 3:** Results of Logistic Regression*, Factors Significantly Associated with Net Use for Children <5, Central and Eastern Regions, Ghana 2004

Characteristic	Children <5 who slept under a bednet in the previous 24 hours	
	
	Odds Ratio	95% CI
Region		
Central	1.0	--
Eastern	4.9	(2.6, 9.2)
Water Supply		
Piped in/Covered well in yard	1.0	--
Public Tap/Borehole	0.6	(0.1, 3.2)
Open Water Source	1.9	(0.4, 10.1)
Food Security		
Food Insecure with Hunger	1.0	--
Food Insecure without Hunger	2.2	(1.0, 5.1)
Food Secure	2.5	(1.2, 4.9)
Causes		
Overworking yourself		
Yes	2.0	(1.1, 3.7)
Protective methods		
Sleeping under ITN		
Yes	1.8	(0.8, 4.0)
Mosquito Coils		
No	1.5	(0.9, 2.4)
Clearing bushes around house		
Yes	1.5	(0.9, 2.6)
Signs and Symptoms		
Diarrhoea		
No	1.9	(0.8, 4.3)
Dizziness		
No	5.6	(1.3, 24.6)
Agree Statement: Child with fever does not need to complete full course of treatment if conditions improve		
Disagree	2.2	(1.0, 4.5)
Who is most vulnerable to malaria		
Adult males (above 18 yrs)		
Yes	2.2	(1.1, 4.5)
Everyone		
Yes	1.8	(1.0, 3.2)
How to protect from malaria during pregnancy		
Keep surroundings clean		
Yes	1.8	(1.1, 3.0)
Don't Know		
Yes	3.5	(0.9, 14.3)

* based on stepwise regression, *p *= .20

## Discussion

Results from this study suggest that region of residence, food security and beliefs about malaria were associated with sleeping under a bednet. For example, doers were nearly five times more likely to come from the Afram Plains District than non-doers. Mothers from food secure households were 2.5 times more likely than their food insecure peers to have children sleep under bednets. Doers also had relatively high knowledge related to malaria; however, misconceptions about malaria and lack of knowledge also existed among doers. More than 90% of respondents identified mosquito bites as a cause of malaria, though mosquito bites were not commonly identified as the only cause. Doers were more likely than non-doers to believe that malaria is caused by overworking oneself and that keeping surroundings clean is protective against malaria during pregnancy. Likewise, both doers and non-doers identified children and pregnant women as the groups most vulnerable to malaria (85% and 60%, respectively). However, doers were more likely than non-doers to also believe that adult males were most vulnerable to malaria. Lastly, doers were 5.6 times more likely than non-doers to believe that dizziness is not a symptom of malaria. Though dizziness is not considered a traditional symptom of malaria it can occur as an atypical manifestation, particularly when severe and acute anaemia results from infection.

This study tested the hypothesis that mothers who practice optimal malaria prevention behaviours by having their child sleep under a bednet (doers) 1) have a better understanding of the cause of malaria as well as appropriate protective methods; 2) are more likely to know the signs and symptoms of malaria; 3) are able to identify those most vulnerable to malaria; 4) know the potential consequences of malaria during pregnancy; and 5) understand how to protect pregnant women from malaria. The results of this study do not support these hypotheses. Logistic regression analyses showed that doers did not express higher knowledge related to protective methods against malaria. Additionally, they were no more likely than non-doers to identify only the correct cause of malaria, the standard signs and symptoms of the disease, or those groups recognised as most vulnerable to the disease. No notable statistical differences were observed between doers and non-doers in relation to knowledge of the consequences of malaria during pregnancy. Though doers were more likely than non-doers to believe that keeping surroundings clean was protective against malaria during pregnancy (which offers some protection by reducing mosquito breeding near the home), they were not more likely to identify the better protective methods of both using ITNs and taking antimalarial drugs.

Greater knowledge about malaria causation, symptoms and prevention as well as groups most vulnerable to malaria does not necessarily translate into improved health behaviour, as evidenced by the fact that in some cases non-doers were more knowledgeable about malaria than doers. Similar to what other researchers have found in Ghana and elsewhere [15,17-20], it appears that "knowledge and behaviour change have no simple and direct relationship, and other intervening and contradicting variables may lead to behaviour consistent and inconsistent with knowledge" (p.376) [9]. The gap between knowledge, attitudes and practices, however, is not a new phenomenon, nor one unique to malaria. Others have noted similar results for other health behaviours and products including the use of personal protective equipment during pesticide application [21], the use of condoms [22,23] and family planning methods [24,25], among others.

This study is subject to a number of limitations. As noted previously, the survey was designed to evaluate the impact of Freedom From Hunger's malaria module on key behaviours related to malaria prevention and control. Consequently, information about a broad array of behavioural determinants such as cost and availability of bednets, attitudes about bednet use, perceptions of malaria disease risk and so on was not collected. In addition, the Eastern and Central regions were substantially different with respect to socio-demographic characteristics, geography and accessibility and availability of health services. Interpreting differences between the two regions is therefore difficult because such differences may reflect variations in health care access, education, income and malaria prevalence. Exposure to educational messages and malaria interventions likely varies between the study areas as well. For example, according to the Demographic and Health Survey (DHS) conducted in Ghana in 2003, households in the Eastern Region reported higher rates of exposure to media messages on malaria compared to households in the Central Region. In addition, the Ministry of Health (MOH) sponsors a bednet programme to improve access for children less than five years of age in the Central Region. In Afram Plains, the United States Agency for International Development (USAID) and the United Kingdom's Department for International Development (DFID) offer vouchers for ITNs. The authors of this paper have not been able to identify which programme has been more successful (the voucher programme in the Eastern region or the MOH programme in the Central Region). However, based on the experience of FFH staff in Ghana, there is some indication that individuals in the Eastern Region (who live in close proximity to the Volta River) are more likely to use bednets as a measure to reduce nuisance from mosquitoes and other insects. Such regional differences in bednet usage among children less than five years of age have also been identified by the 2003 DHS. Results from the DHS show the Central Region as that having the lowest rates of bednet ownership and usage among children under age five in the country, with the Eastern Region having just slightly higher rates in both cases [26].

Results from this study are comparable to findings from other studies conducted in various regions of Ghana. For example, the NetMark project reports that 82% of surveyed Ghanaians identified mosquitoes as a cause of malaria and 65% correctly identified fever as a symptom of malaria [27]. Likewise, Adongo and colleagues found that 79% of respondents identified mosquitoes as the cause of malaria; however, alternative cultural explanations – including exposure to heat – were also common. In addition, 92% of respondents believed that bednets could prevent malaria [9]. Agyepong and Manderson found that the most commonly identified signs and symptoms of malaria among children included hot body (fever), yellow eyes and urine, vomiting and refusal to feed [13].

## Conclusion

Findings from this study and research carried out by others suggest that many Ghanaians fail to use bednets and insecticide treated nets in particular. While a majority of individuals in this study possess at least some level of correct knowledge, alternative beliefs and attitudes related to disease causation, prevention and treatment persist and appear to affect the adoption of ideal health behaviours. In the words of Agyepong and Manderson, "people's ability to comply with interventions and to treat sickness is affected by their acceptance of the interventions, their understanding of the nature of the illness and the relationship between vector and infection, and other social, economic and cultural factors" (p.79) [10].

When developing interventions for populations with traditional beliefs and culturally defined behaviours, it is useful to classify such beliefs and behaviours as positive, harmful, or neutral. Programme planners and implementers should reinforce positive beliefs and behaviours and identify culturally appropriate means for modifying harmful beliefs and behaviours. Neutral beliefs and behaviours can be left alone or, if needed, incorporated into health messages that promote ideal health behaviours. Though such culturally based ideas and behaviours may vary somewhat between communities and efforts to identify and include them in health education messages can add to the initial cost of programmes and interventions, the benefit of the added effectiveness over time cannot be discounted.

In Ghana, caregivers maintain a number of positive beliefs and practice behaviours conducive to the prevention of malaria. The majority of individuals in this study were able to recognize that mosquitoes play a role in malaria transmission and that bednets can protect against disease. Results presented here did not highlight any beliefs about malaria that were outright harmful; however, participants did possess some neutral beliefs that could be used to support positive beliefs and practices. For example, the notion that malaria is caused by exposure to heat or overworking oneself can be classified as a neutral belief – one that has traditionally served to explain the effects of malaria on the blood as well as the visible symptoms of the disease [14]. Combining alternative, culturally based ideas about malaria causation – including exposure to heat – with biomedical explanations of disease transmission may improve the uptake of educational messages and encourage the adoption of appropriate preventive behaviours [9]. Programme planners may wish to develop messages that suggest that mosquitoes carrying the disease prefer to feed on blood that has been heated by long hours working or playing in the sun. Bednets could then be promoted as a way of keeping mosquitoes from detecting and feeding on hot blood at night, particularly during the dry season when net use typically declines and complaints about nets being too hot increase. It is important that programme planners use local terminology for diseases that correspond closely to the clinical definition of malaria. Additionally, while this particular study did not assess issues of cost and access, there is evidence to suggest that programmes and policies should continue to improve the availability of ITNs for the poorest income groups and those most affected by malaria [7].

In Ghana, malaria remains an important cause of childhood morbidity and mortality. The use of bednets – and in particular ITNs – is an effective method for reducing the incidence and disease burden of malaria. Despite high levels of knowledge about malaria, bednet use remains inadequate if the lives of vulnerable Ghanaian children are to be protected. Efforts to improve the use of bednets must extend beyond sharing information about the benefits of ITN use. Such efforts must address sociocultural, economic and other determinants of behaviour. Educational messages must be culturally sensitive and capitalize on the positive beliefs and behaviours that already exist in local communities. Likewise, programmes that mobilize communities can play a critical role in the adoption of preventive behaviours and improved rates of child survival.

## Authors' contributions

ND participated in data analysis and interpretation, prepared the manuscript and was responsible for revision and submission of the manuscript. BC performed data analysis and interpretation and participated in manuscript revision. KD was involved in research design, data analysis and interpretation and manuscript revision. NI was also involved in research design and manuscript preparation. BG participated in research design and manuscript revision. SA provided guidance on data analyses and was involved in the interpretation of data and manuscript revision. RD participated in research design and manuscript revision as well. All authors read and approved the final manuscript.

## Supplementary Material

Additional File 1 Regional Differences, Study of Knowledge, Attitudes and Practices of Malaria Prevention, Central & Eastern Regions, Ghana 2004. This table shows the observed differences in demographics and malaria related knowledge (based on bivariate analyses) between respondents in the Eastern and Central regions. Click here for file
