# Supplementary material for: Who sleeps under bednets in Ghana? A doer/non-doer analysis of malaria prevention behaviours
Source: Malar J. 2006 Jul 25;5:61. doi: 10.1186/1475-2875-5-61 (PMC1553454; doi:10.1186/1475-2875-5-61)
Supplement: Additional File 1 — Regional Differences, Study of Knowledge, Attitudes and Practices of Malaria Prevention, Central & Eastern Regions, Ghana 2004. This table shows the observed differences in demographics and malaria related knowledge (based on bivariate analyses) between respondents in the Eastern and Central regions. [file 1475-2875-5-61-S1.doc]

Regional Differences, Study of Knowledge, Attitudes and Practices of Malaria Prevention, Central & Eastern Regions, Ghana 2004

| Characteristic | Central | Eastern |  |
| --- | --- | --- | --- |
|  | N=537  % | N=518  % | *p* |
|  |  |  |  |
| Respondent's Age |  |  | **.0259** |
| 15-24 | 15.1 | 17.9 |  |
| 25-34 | 40.8 | 46.3 |  |
| 35-44 | 32.4 | 28.2 |  |
| 45-49 | 11.7 | 7.6 |  |
| Belong to a credit association | 49.9 | 48.3 | .5910 |
| Highest level of schooling attended |  |  | **.0025** |
| No school | 15.5 | 21.2 |  |
| Primary | 20.8 | 26.3 |  |
| Junior secondary/middle | 56.6 | 45.3 |  |
| Senior secondary/ vocational/ higher | 7.1 | 7.3 |  |
| Household Size |  |  | **<.0001** |
| 1-3 | 9.5 | 17.3 |  |
| 4-5 | 27.8 | 42.2 |  |
| 6-7 | 37.4 | 27.6 |  |
| 8 or more | 25.3 | 12.8 |  |
| Religion |  |  | **.0054** |
| Christian | 91.9 | 86.6 |  |
| Muslim / Other | 8.1 | 13.4 |  |
| Water Supply |  |  | **<.0001** |
| Piped in/Covered well in yard | 7.6 | 0.6 |  |
| Public Tap / Borehole | 69.5 | 86.7 |  |
| Open Water Source | 22.9 | 12.7 |  |
| Toilet Facility |  |  | **<.0001** |
| Public Latrine | 72.1 | 43.2 |  |
| Private Toilet/Latrine | 22.7 | 39.7 |  |
| Bush | 5.2 | 17.2 |  |
| Received antenatal care during last pregnancy | 98.8 | 97.0 | **.0442** |
| Received professional antenatal care during last pregnancy | 99.4 | 96.3 | **.0007** |
| Food Security |  |  | **.0152** |
| Food Secure | 52.3 | 59.8 |  |
| Food Insecure without Hunger | 24.4 | 17.5 |  |
| Food Insecure with Hunger | 23.2 | 22.6 |  |
| Believe mosquito bites cause malaria | 92.9 | 97.3 | **.0011** |
| Believe staying in the sun too long causes malaria | 36.4 | 46.4 | **.0010** |
| Believe working too close to fire causes malaria | 14.0 | 15.4 | .1000 |
| Believe overworking yourself causes malaria | 14.4 | 13.5 | .5198 |
| Believe in spiritual/superstitious causes of malaria | 1.3 | 2.7 | .6685 |
| Believe in other causes of malaria | 26.1 | 13.8 | **<.0001** |
| Do not know what causes malaria | 2.1 | 1.2 | .2577 |
| Believe in the correct cause of malaria (mosquito bites only) | 33.5 | 26.0 | **.0083** |
| Believe sleeping under an ITN is protective against malaria | 60.5 | 93.8 | **<.0001** |
| Believe mosquito coils are protective against malaria | 59.9 | 58.9 | .7370 |
| Believe clearing bushes around the house is protective against malaria | 52.4 | 46.4 | .0508 |
| Believe wearing clothes to cover the body is protective against malaria | 8.0 | 9.9 | .2767 |
| Believe disturbing mosquito hiding places is protective against malaria | 6.3 | 11.7 | **.0024** |
| Believe in other protective methods against malaria | 25.4 | 5.7 | **<.0001** |
| Do not know protective methods against malaria | 3.4 | 1.2 | **0.0178** |
| Believe that the best way to prevent malaria is: |  |  | **<.0001** |
| Using an ITN | 61.5 | 93.0 |  |
| Other good alternatives | 27.9 | 2.9 |  |
| Don’t know | 10.6 | 4.1 |  |
| Believe fever is a sign/symptom of malaria | 68.8 | 67.5 | 0.6273 |
| Believe vomiting is a sign/symptom of malaria | 42.0 | 70.8 | **<.0001** |
| Believe yellowish/dark urine is a sign/symptom of malaria | 58.0 | 41.3 | **<.0001** |
| Believe a lack of appetite is a sign/symptom of malaria | 39.0 | 40.4 | 0.6530 |
| Believe chills is a sign/symptom of malaria | 27.6 | 16.0 | **<.0001** |
| Believe diarrhoea is a sign/symptom of malaria | 17.2 | 10.7 | **.0027** |
| Believe headache is a sign/symptom of malaria | 19.2 | 8.4 | **<.0001** |
| Believe joint pain or bone pain is a sign/symptom of malaria | 12.3 | 10.9 | 0.4804 |
| Believe dizziness is a sign/symptom of malaria | 10.1 | 6.4 | **0.0325** |
| Believe constipation is a sign/symptom of malaria | 2.6 | 3.5 | 0.3985 |
| Believe in other signs/symptoms of malaria | 21.6 | 7.8 | **<.0001** |
| Believe that a child with fever needs to complete a full course of treatment even if his conditions improve | 80.3 | 82.5 | 0.3821 |
| Believe that children <5 are most vulnerable to malaria | 83.4 | 88.5 | **0.0176** |
| Believe that children 6-14 years old are most vulnerable to malaria | 20.0 | 6.6 | **<.0001** |
| Believe that pregnant women are most vulnerable to malaria | 49.8 | 64.9 | **<.0001** |
| Believe that women of reproductive age (15-49) are most vulnerable to malaria | 14.2 | 4.5 | **<.0001** |
| Believe that adolescent boys (15-18 yrs) are most vulnerable to malaria | 6.3 | 4.5 | 0.1841 |
| Believe that adult males (above 18 yrs) are most vulnerable to malaria | 9.3 | 8.0 | 0.4421 |
| Believe that everyone is most vulnerable to malaria | 12.9 | 18.3 | **0.0149** |
| Do not know who is most vulnerable to malaria | 2.8 | 1.2 | 0.0597 |
| Believe that only children <5 years are most vulnerable to malaria | 20.5 | 10.7 | **<.0001** |
| Believe that only pregnant women are most vulnerable to malaria | 1.9 | 2.9 | 0.2613 |
| Believe that only children <5 and pregnant women are most vulnerable | 22.2 | 29.4 | **0.0074** |
| Believe that malaria during pregnancy can lead to miscarriage/abortion | 67.2 | 56.3 | **0.0003** |
| Believe that malaria during pregnancy can lead to death | 65.1 | 52.4 | **<.0001** |
| Believe that malaria during pregnancy can lead to premature delivery | 26.9 | 45.6 | **<.0001** |
| Believe that malaria during pregnancy can lead to anaemia | 25.2 | 11.7 | **<.0001** |
| Believe that malaria during pregnancy can lead to profuse bleeding | 11.2 | 17.7 | **0.0025** |
| Believe that malaria during pregnancy can lead to something else | 3.4 | 4.7 | 0.2756 |
| Do not know any consequences of malaria during pregnancy | 1.1 | 5.5 | **<.0001** |
| Believe that starting antenatal care once pregnant is protective against malaria during pregnancy | 81.7 | 51.5 | **<.0001** |
| Believe that sleeping under an ITN is protective against malaria during pregnancy | 29.7 | 61.6 | **<.0001** |
| Believe that eating good food/a balanced diet is protective against malaria during pregnancy | 50.9 | 40.9 | **0.0012** |
| Believe that keeping surroundings clean is protective against malaria during pregnancy | 25.2 | 33.1 | **0.0046** |
| Believe that taking antimalarials as directed is protective against malaria during pregnancy | 28.0 | 9.2 | **<.0001** |
| Believe that treating worm infestations is protective against malaria during pregnancy | 3.0 | 6.2 | **0.0117** |
| Believe that something else is protective against malaria during pregnancy | 13.3 | 4.9 | **<.0001** |
| Do not know of any ways to protect against malaria during pregnancy | 1.3 | 1.6 | 0.7296 |
| Believe that pregnant women can have malaria without knowing or showing signs of it | 58.9 | 46.2 | **<.0001** |
|  |  |  |  |
